# Efficiency of Vitamin D Supplementation in Healthy Adults is Associated with Body Mass Index and Baseline Serum 25-Hydroxyvitamin D Level

**DOI:** 10.3390/nu12051268

**Published:** 2020-04-29

**Authors:** Katja Žmitek, Maša Hribar, Hristo Hristov, Igor Pravst

**Affiliations:** 1 Nutrition Institute, Tržaška cesta 40, SI-1000 Ljubljana, Slovenia; masa.hribar@nutris.org (M.H.); hristo.hristov@nutris.org (H.H.); igor.pravst@nutris.org (I.P.); 2 VIST–Higher School of Applied Sciences, Gerbičeva cesta 51A, SI-1000 Ljubljana, Slovenia; 3 Department of Food Science and Technology, Biotechnical Faculty, University of Ljubljana, Jamnikarjeva 101, 1000 Ljubljana, Slovenia

**Keywords:** vitamin D3, cholecalciferol, absorption, deficiency, supplementation, buccal spray, capsules

## Abstract

Vitamin D (VitD) has a critical role in phosphorous–calcium metabolism as well as an important role in the immune system. In the human body, VitD is synthesized as cholecalciferol in the skin, but this process requires sunlight (UVB) radiation. Numerous reports showed high prevalence of VitD deficiency, particularly during the winter season, indicating the importance of VitD supplementation. Various factors can affect the absorption of VitD, including dosage and formulation. The primary study objective was to examine the efficiency of supplementation with three different formulations containing cholecalciferol in comparison with the control group. The secondary objective was to identify other factors affecting increase in serum 25-OH-VitD. A randomized controlled intervention study was conducted in Slovenia during wintertime (January– March) on 105 apparently healthy subjects (aged 18–65 years) with suboptimal VitD status (25-OH-VitD 30–50 nmol/L). Subjects were randomized into four groups: three treatment groups receiving (A) capsules with starch-adsorbed VitD, (B) oil-based Valens VitD oral spray, or (C) water-based Valens VitD oral spray and a control group (D) which did not receive supplemental VitD. Two months of supplementation with cholecalciferol (1000 IU; 25 µg daily) resulted in significant increase in serum 25-OH-VitD levels in comparison with control group (pooled Δc 32.8 nmol/L; 95% CI: 23.0, 42.5, *p* < 0.0001). While we did not observe any significant differences between the tested formulations, the efficiency of supplementation was associated with body mass index and baseline serum 25-OH-VitD level. Higher supplementation efficiency was observed in participants with normal body weight (BMI < 25) and in those with more pronounced VitD insufficiency. We also determined that tested dosage was not sufficient to achieve recommended 25-OH-VitD levels in all subjects.

## 1. Introduction

Vitamin D is a prohormone with various functions in the human body. Sufficient vitamin D levels are essential to support musculoskeletal health and a range of other conditions. While vitamin D is needed for normal absorption of minerals (particularly calcium), supporting normal bone development and optimal bone mineral density, vitamin D deficiency might also be connected with some chronic or acute illnesses, including infectious diseases and type 2 diabetes mellitus, and is also linked to decreased immunity and some neurocognitive disorders [1,2].

It is well established that vitamin D deficiency is a worldwide problem [3], present both in developed and less developed countries. Vitamin D in the body originates from both biosynthesis in the skin triggered by ultraviolet B (UVB) irradiation as well as dietary sources. Sunlight exposure, rather than diet, has been reported as the main source for the majority of the population, while in the absence of sufficient sun exposure, vitamin D becomes an essential nutrient [4,5]. In human skin, UVB radiation initiates vitamin D3 synthesis through the conversion of 7-dehydrocholesterol (7-DHC) to previtamin D3. A heat isomerization of previtamin D3 to cholecalciferol (vitamin D3) is followed by hydroxylation in the liver to 25-hydroxyvitamin D (25-OH-VitD) and further in kidney to the active form of the vitamin 1,25-dihydroxyvitamin D3 (1,25-OH-VitD) [6].

Vitamin D sufficiency is usually determined by serum 25-OH-VitD levels. While there are different views about optimal plasma 25-OH-VitD levels [5], it is clear that serum levels below 30 nmol/L are presenting a risk for rickets and/or osteomalacia and should be avoided at all ages. Typically, vitamin D deficiency is marked by a threshold of less than 30 nmol/L and insufficiency by concentrations in the range 30–50 nmol/L [5,7,8,9], although some experts advocate higher sufficiency thresholds. For example, considering the available evidence on skeletal and extra-skeletal effects of vitamin D, the Endocrine Society established 75 nmol/L as the target concentration for optimal vitamin D level [10,11,12].

Seasonal variation of serum 25-OH-VitD concentrations has been observed in the northern hemisphere, and quite high prevalence of vitamin D deficiency is reported in several countries [5,13,14]. Considering that sunlight-induced vitamin D synthesis is not effective during wintertime, substantial proportions of the European population rely on dietary vitamin D and after-summer body stores to maintain vitamin D status. European Food Safety Authority set adequate intake of vitamin D (in the absence of sunlight-induced biosynthesis) at the level of 15 μg daily [15], while daily tolerable upper intake level (UL) for adults was set at 100 µg (4.000 IU) [16]. While on one hand many people are at risk for vitamin D deficiency, self-administration of high doses of vitamin D in the general population can also result in dangerously high 25-hydroxyvitamin D concentrations [17].

Surprisingly, there is not much reliable data on factors that affect absorption of vitamin D [18]. In a very recent review, Mazahery et al. highlighted baseline 25-OH-VitD, body mass index (BMI), dose, and type of vitamin D as most probable factors affecting bioavailability of ingested vitamin D [19]. Even more recently, Grammatikopoulou et al. performed a systematic review of randomized controlled trials (RCT), investigating inconsistencies in the efficacy of novel delivery forms of vitamin D [20], noting the need for additional RCTs. While some studies reported higher absorption efficacy of spray formulations, other did not observe such effects.

Our primary objective was to examine efficiency of supplementation with three different formulations containing cholecalciferol–vitamin D3 (starch-adsorbed vitamin D, oil-based oral spray, and water-based oral spray) in comparison with control group. The secondary objective was to identify other factors affecting supplementation-related increase in serum 25-OH-VitD levels. The intervention was daily supplementation with 25 µg (1000 IU) of vitamin D3 for eight weeks.

## 2. Experimental Section

### 2.1. Study Design

The study employed a single-center, randomized, controlled, parallel design. Blinding was not possible because of different forms of the test products—some products were capsules and others were sprays; the experiment was therefore conducted as open labeled. The study was in full compliance with the principles laid out in the Declaration of Helsinki. The study protocol was approved by the Ethics Committee of the Higher School of Applied Sciences (Approval No. 2018/4-ET-SK) and included in the ClinicalTrials.gov register under record NCT03810261.

### 2.2. Study Population

Invitation to participate in the study was published on the institutional web site of the Nutrition Institute (Slovenia) and social media. Inclusion criteria were Caucasian race, age between 18 and 65 years, willingness to avoid consumption of any additional food supplements containing vitamin D during the study, willingness to avoid use of tanning beds or other artificial UVB sources, willingness to follow all study procedures, and suboptimal serum 25-OH-VitD concentration (30–50 nmol/L). Exclusion criteria were pregnancy or breastfeeding; known or suspected allergy to any ingredient of the tested products; pronounced avoidance of sunshine (e.g., reporting of allergy to the sun); use of food supplements containing vitamin D, fish oil, or omega 3 fatty acids in the last three months prior to inclusion; special dietary habits (veganism, low-carb high-fat (LCHF) diet, and caloric restriction diet; note: vegetarians were not excluded); on a diet prescribed by the medical profession; current disorders of the kidneys, thyroid, digestive tract, osteoporosis and other bone diseases, and skin diseases; other diseases and conditions that affect the absorption and synthesis of vitamin D; and exposure to stronger sunlight in the last three months prior to fulfilment of an inclusion survey (travel to countries with stronger sunshine and use of tanning beds). Serum 25-OH-vitD levels are also affected by vitamin D intake with ordinary foods. To minimize the effect of this factor, regular (daily) consumption of vitamin D-enriched foods, such as margarine or plant substitutes for milk/milk products was also an exclusion criteria.

To enable sample size calculation (study power 0.9, alpha 0.05), a preliminary pilot study was conducted on 10 subjects (unpublished data) and intersubject variability in serum 25-OH-VitD concentration (SD) was determined in the population with suboptimal vitamin D status, together with expected increase of serum vitamin D level after two months of supplementation for water-based vitamin D oral spray. It was determined that at least 18 subjects per study group are needed to detect 35% difference in increase of serum 25-OH-VitD concentration between two study groups (SD = 2.5; *p* > 0.05). To compensate for possible dropouts among the winter period, we decided to enlarge the study sample to at least 20 subjects per group.

**Figure 1:**
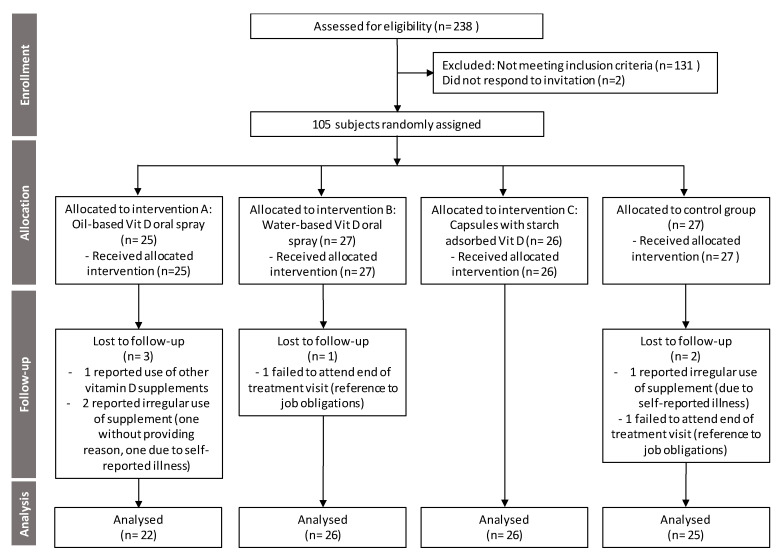
CONSORT (Consolidated Standards of Reporting Trials) flow diagram showing trial design and subjects’ assignment and progression through the trial.

A CONSORT (Consolidated Standards of Reporting Trials) flow diagram showing trial design and subjects’ assignment and progression through the trial is presented in Figure 1. Initially, eligibility of 238 healthy adults was screened for 25-OH-VitD serum levels, ranging in age from 18 to 64 years. Among those, 107 subjects with suboptimal serum 25-OH-VitD concentration were invited to collaborate in the trial, two of those did not respond, and therefore, 105 were enrolled in the trial and assigned to one of four groups using stratified randomization by gender and baseline 25-OH-VitD levels. Trial was conducted in Slovenia during wintertime; the study started in January 2019 (signed consent, screening and baseline serum 25-OH-VitD concentration, randomization, and start of treatment) and ended in March 2019 (compliance assessment and measurement of serum 25-OH-VitD concentration). Consistent with the principles laid down in the Declaration of Helsinki, all subjects provided signed informed consent before recruitment. No adverse events were reported.

### 2.3. Study Products and Intervention

The trial included three treatment groups receiving 25 µg (1000 IU) of cholecalciferol per day for 8 weeks and a control group not supplemented with vitamin D:

- Group A: capsules with starch-adsorbed vitamin D;
- Group B: oil-based Valens vitamin D oral spray;
- Group C: water-based Valens vitamin D oral spray; and
- Group D: control group.

All three tested products are commercially available formulations containing cholecalciferol; vitamin D content in the test products was verified in an independent accredited laboratory (Chelab S.r.l, Resana, Italy).

### 2.4. Serum 25-OH-VitD Concentration

Serum 25-OH-VitD concentration was measured before intervention (January) and at the last day of the treatment (March). Following an overnight fast, a blood sample was taken the next morning. Blood collection and analyses were done in accredited medical diagnostic laboratory Adrialab/Synlab (Ljubljana, Slovenia) using standard chemiluminescent method. Architect 25-OH vitamin D (Abbott Ireland, Longord, Ireland) chemiluminescent microparticle immunoassay was used for quantitative determination of 25-OH vitamin D in human serum. Correlation coefficient with ID-LC-MS/MS method within assay’s measuring interval (12–378 nmol/L) is r = 0.99 (95% CI: 0.99, 1.05).

### 2.5. Data Processing and Analyses

The data were processed and analyzed using Stata Statistical Software, Release 15 (StataCorp LLC, College Station, TX, USA), Microsoft Excel (Microsoft Corp., version 16.0.12325.20280, Redmond, WA, USA), and GraphPad Prism (GraphPad Software, version 8.2.0., San Diego, CA, USA). Descriptive characteristics as well as the proportions of participants with different levels of 25-OH-VitD are presented for all participants and per treatment. Analyses were conducted using the per-protocol principle. For a comparison of vitamin D changes between different formulation and control, a two-way ANOVA with Bonferroni’s multiple comparisons post hoc test was used. Logistic regression analysis was used to determine the significant differences in the mean increase in serum 25-OH-VitD levels between different subpopulations of the samples. Multivariable logistic regression analysis was undertaken with selected parameters (baseline 25-OH-VitD concentration, intervention type, gender, age, and BMI)) to determine independent predictors of above mean increase in serum 25-OH-VitD level (Δc > 29.0 nmol/L). Analyses were done for all participants in the treatment groups (A + B + C) with a missing BMI value for one subject. In addition, Pearson linear correlation was used to determine the significant relationship between BMI and treatment-related increase in serum 25-OH-VitD concentration. The significance level was set at 0.05.

## 3. Results

**Figure 2:**
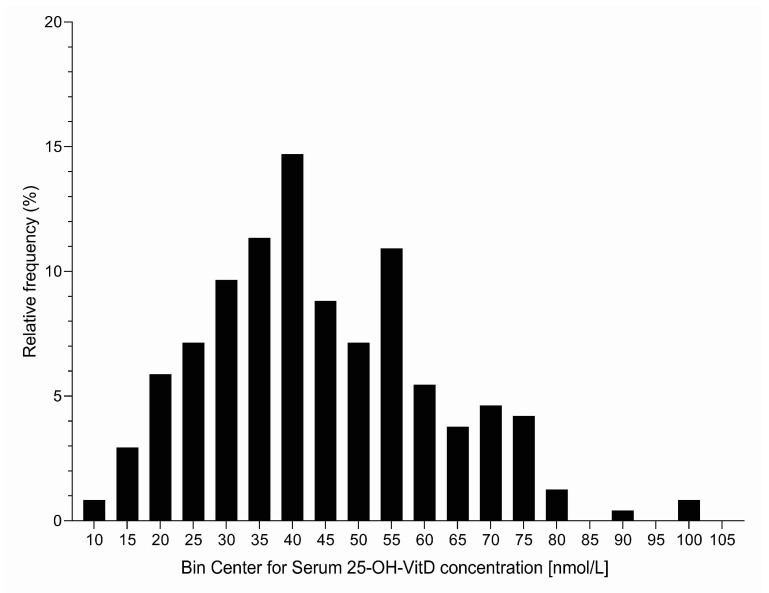
Histogram presenting distribution of wintertime serum 25-OH-Vitamin D concentrations in subjects included to screening (N = 238, February).

A total of 238 healthy adults were screened for serum 25-OH-VitD concentration (Table 1 and Figure 2), ranging in age from 18 to 64 years (mean age 37.7 ± 11.4 (SD)); about half of those were male and half were female. Altogether, 49 subjects (21%; 18% for males and 23% for females) had serum 25-OH-VitD levels below 30 nmol/L. These were not invited to the intervention phase (not meeting inclusion criteria) because it would not be ethical to have them in a control group without vitamin D supplementation. On the other hand, 107 subjects (45%) had serum 25-OH-VitD levels from 30 to up to 50 nmol/L, with some minor differences between gender and different age groups (Table 1). These were meeting our inclusion criteria for invitation to the intervention phase. Interestingly, only 82 subjects (34% of the whole sample) had sufficient vitamin D status (serum 25-OH-VitD levels above 50 nmol/L). The proportion of vitamin D-sufficient subjects was higher in females (37%) than in males (31%).

In line with the protocol, all one hundred and seven subjects with serum 25-OH-VitD concentration in the range from 30 to > 50 nmol/L were invited to the study. Of these, 105 subjects responded and were therefore enrolled to the study. The study was conducted as planned without protocol amendments. Six subjects were lost to follow up, while 99 (94%) completed the study. Control and treatment groups are more detailly described in Table 2.

**Figure 3:**
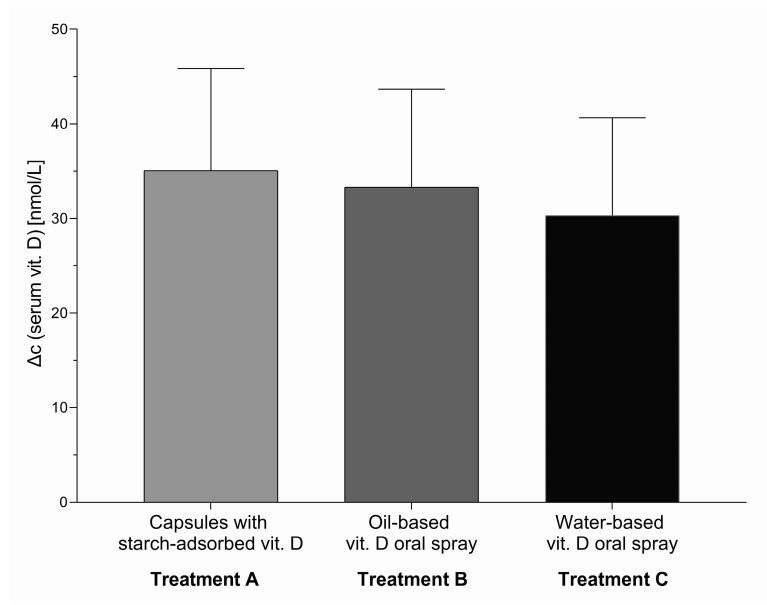
Treatment effect (change in serum 25-OH-vitD concentration in comparison with the control group D) of the intervention (8 weeks of daily supplementation with 1000 IU; 25 µg vitamin D) for three tested vitamin D formulations in comparison to control group, with 95% CI: The effect was statistically significant (*p* < 0.0001) in all three intervention groups.

To address the primary study objective, we first calculated the difference in serum 25-OH-VitD concentration after the intervention in comparison with control group. Two months of supplementation with vitamin D (1000 IU; 25 µg daily) during winter resulted in considerable increase in average serum vitamin D levels in comparison with control group. A significant treatment effect was observed in all three treatment groups (A, B, and C) with differences in serum 25-OH-VitD concentration for 35.0 nmol/L (95% CI 24.3, 45.8, *p* < 0.0001), 33.3 nmol/L (95% CI 23.0, 43.8, *p* < 0.0001), and 30.5 nmol/L (95% CI 20.0, 40.8, *p* < 0.0001), respectively (Figure 3). Considering that there were no significant differences in the treatment effect between groups A, B, and C, we also calculated the pooled treatment effect for all three treatment groups (A + B + C), which was also significant (Δc = 32.8 nmol/L; 95% CI: 23.0, 42.5, *p* < 0.0001).

**Figure 4:**
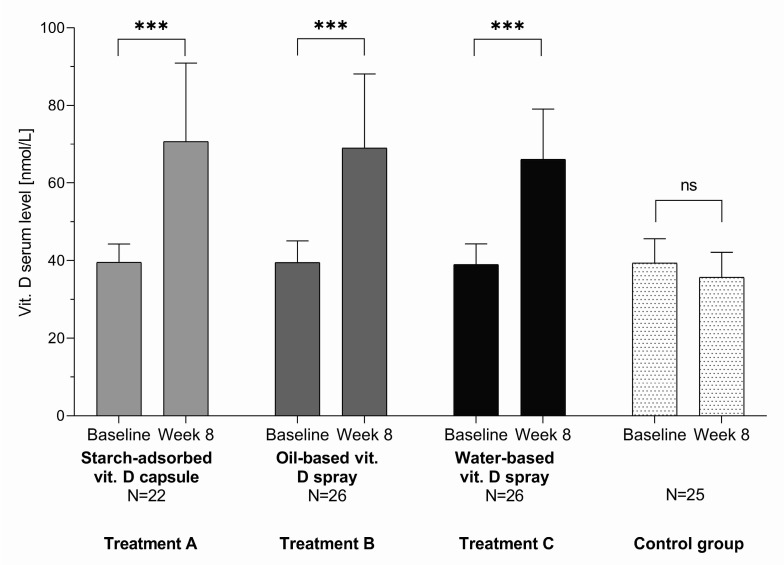
Serum 25-OH-VitD levels at baseline and after 8 weeks of vitamin D supplementation with three different vitamin D formulations in test groups in comparison to control group; *** *p* < 0.0001 for change from baseline within group; ns: not significant.

Comparison of serum 25-OH-vitD concentrations before and after 8 weeks of vitamin D supplementation within groups shows that, in group A receiving capsules with starch-adsorbed vitamin D, it increased from 39.5 ± 4.8 (mean ± SD) to 70.8 ± 20.3 nmol/L; in group B receiving oil-based vitamin D oral spray, it increased from 39.5 ± 5.5 to 69.0 ± 19.3 nmol/L; and in group C receiving water-based vitamin D oral spray, it increased from 39.0 ± 5.3 to 66.0 ± 13.0 nmol/L (*p* < 0.0001 for all three treatment groups). Looking at all subjects in all three treatment groups, serum 25-OH-vitD level increased from 39.3 ± 5.5 to 68.5 ± 17.5 nmol/L. On the other hand, in the control group (D), we observed minor nonsignificant decrease in serum 25-OH-vitD levels from January to March (from 39.5 ± 6.3 to 35.8 ± 6.5 nmol/L; *p* = 0.133) (Figure 4).

Considering that we did not observe significant differences between the three tested formulations, a combined treatment sample (groups A + B + C) was used for exploratory data analyses, providing insights into parameters influencing the increase in serum 25-OH-vitD level after the intervention. Multivariable logistic regression analyses were done using intervention type, baseline 25-OH-VitD concentration, gender, age and BMI as possible independent predictors of increase in serum 25-OH-VitD levels in subjects supplemented with vitamin D. Mean increase in serum 25-OH-VitD concentration in subjects in all three treatment groups (A + B + C; N = 74) was 29.2 nmol/L (95% CI: 25.5, 32.8, *p* < 0.0001). Table 3 presents the prevalence of an increase in serum 25-OH-VitD level above this mean and adjusted odds ratios (95% CI). Subjects with lower (pre-intervention) baseline 25-OH-VitD concentration and those with lower body mass index (BMI < 25) were more likely to have an above-mean increase in serum 25-OH-VitD level (*p* = 0.0474 and *p* < 0.0001, respectively). On the other hand, intervention type (capsules, oil-based spray, and water-based spray), gender, and age were not determined to have a significant effect.

**Figure 5:**
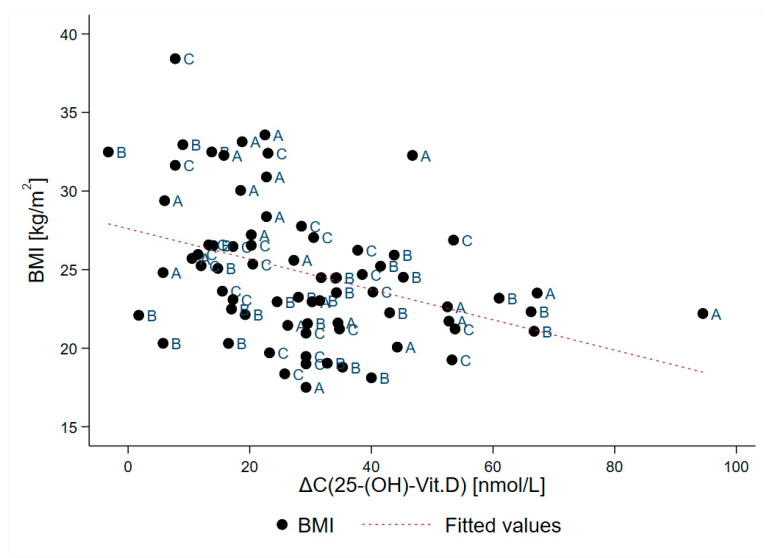
Pearson linear correlation of the treatment-related increase in serum 25-OH-VitD concentration (Δc; nmol/L) and body mass index (BMI; kg/m^2^) for subjects in all three treatment groups (A + B + C; N = 73; missing BMI value for one subject). A—capsules; B—oil-based spray; and C—water-based spray.

**Figure 6:**
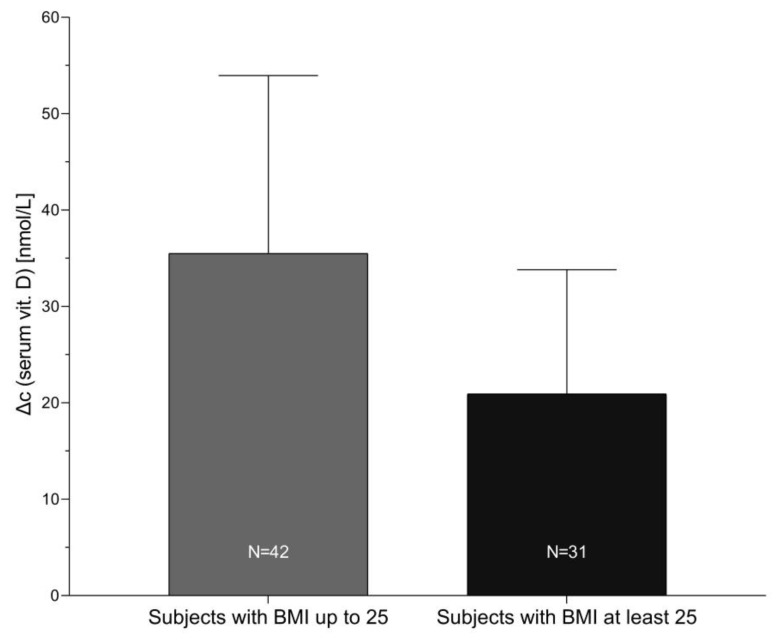
Increase in serum 25-OH-VitD concentration from baseline after 8 weeks of vitamin D supplementation in subgroups of subjects with lower and higher body mass index (BMI; kg/m^2^).

A plot of after-treatment increase in serum 25-OH-VitD concentration in correlation with body mass index is presented in Figure 5. Pearson linear correlation showed negative association (r = −0.385; *p* < 0.001) between increase in the serum 25-OH-VitD concentration and BMI. Sample segmentation showed mean increase in serum 25-OH-VitD concentration for 35.5 ± 18.5 nmol/L in subjects with BMI up to 25 kg/m^2^ (N = 43), while notable lower increase was found in obese/overweight subjects (21.0 ± 13.0 nmol/L; N = 31), as can be observed in Figure 6.

Although the above-reported mean increase in serum 25-OH-VitD levels is more meaningful, we also checked the proportion of subjects that remained vitamin D insufficient after 8 weeks of intervention. As expected, for all subjects (100%) in the control group, the serum 25-OH-VitD level remained vitamin D insufficient, with serum 25-OH-VitD concentrations below 50 nmol/L. On the other hand, in treatment groups (A + B + C), 11% subjects were still below 50 nmol/L while 72% were below 75 nmol/L. The highest observed individual serum 25-OH-VitD concentration after the intervention was 134.0 nmol/L in one subject, while all other subjects had levels below 110 nmol/L.

## 4. Discussion

Different approaches are being used around the globe to address the high incidence of insufficient vitamin D status among populations. While some countries implemented food fortification [21], the most common approach is supplementation of diet with vitamin D preparations. Understanding factors affecting efficiency of the supplementation is therefore crucial. Surprisingly, this topic has received limited scientific attention and there are inconsistencies in the reported results. For example, while cholecalciferol (Vitamin D3) and ergocalciferol (Vitamin D2) were historically considered equipotent, a conflicting evidence has led to uncertainty as to whether both forms are indeed equally efficacious in improving vitamin D status [22,23,24,25,26]. Also, a series of other factors could affect the efficiency of vitamin D absorption, including food matrix and formulation. While there are very limited studies that have compared the influence of the vehicle substance on bioavailability of vitamin D, it has been suggested that, in healthy subjects, cholecalciferol in an oil vehicle is more efficient in comparison to powder formulations [27]. On the other hand, improved absorption of cholecalciferol in a water-based buccal spray was showed in comparison with oil-based soft gelatin capsules, both in healthy subjects and in patients with intestinal malabsorption [28].

Considering the abovementioned inconsistencies, our primary objective was to determine the efficiency of three different vitamin D formulations in treating suboptimal vitamin D status in adult population. Slovenia is a Central European country, and its latitude (45°–46°N) ranks it amongst the countries with the expected high seasonal variations in serum 25-OH-VitD concentrations due to lower intensity of UVB radiation during winter. This study was therefore conducted during wintertime (January–March) when minimal skin biosynthesis of vitamin D was expected. The results obtained for the control group confirmed this; serum 25-OH-VitD concentrations observed in March (35.8 ± 6.5 nmol/L) were even lower than in January (39.5 ± 6.3 nmol/L), although not significantly. Additionally, all 25 subjects in the control group (group D) remained within the interval of insufficient vitamin D status (serum 25-OH-VitD level from 30 to > 50 nmol/L) throughout the study period.

In our trial, capsules with starch-adsorbed vitamin D, oil-based oral spray, and water-based oral spray were used for intervention. Even though cholecalciferol absorption is not majorly affected by the presence or absence of food [29], subjects were advised to consume the supplement with a meal. All three formulations significantly increased serum 25-OH-VitD levels, but in contrast to some previously mentioned reports [27,28], we did not observe significant differences between the tested formulations, although we employed very different matrixes. After eight weeks of supplementation with 25 µg (1000 IU) of cholecalciferol, we observed a mean increase in serum 25-OH-VitD concentration of 29.2 nmol/L (95% CI: 10.2,13.1, *p* < 0.0001). Considering that the total dosage of cholecalciferol administered during the study was 1400 µg (78.400 IU), about 48 µg (1.920 IU) of cholecalciferol was needed to increase serum 25-OH-VitD levels per 1 nmol/L. Comparison of study results with results of other studies is very difficult because studies were conducted with very different designs and because increase of plasma 25-OH-vit D can be affected by a number of factors, for example, study time, race of participants, lifestyle and genetic factors, etc. Penagini et al. investigated efficiency of supplementation with cholecalciferol in children with neurodisabilities (5–17 years) and reported equal effects of two tested formulations [30], noting that buccal spray was more acceptable by the patients. Furthermore, Todd et al. conducted a randomised, open-label, crossover study in which they compared absorption of cholecalciferol from capsule and oral spray solution in healthy adults during wintertime [31]. Their intervention with 3000 IU (75 µg) cholecalciferol per day for 4 weeks showed that the oral spray is an equally effective alternative to capsule supplementation. Treatment resulted in increased serum 25-OH-VitD concentrations for about 28 nmol/L, but it should be noted that the treatment period was substantially shorter than in our study while treatment dosage was much higher (300% of our daily dosage), and therefore, the total dosage of cholecalciferol administered during study (2100 µg; 117.600 IE) was 50% higher than in our case. Nevertheless, about 116 µg (4.640 IU) of cholecalciferol was needed to increase serum 25-OH-VitD levels per 1 nmol/L. Additionally, Williams et al. very recently reported results of a study in which they compared sublingual sprays and capsular vitamin D preparations [32]. They were also using 75 µg (3000 IU) of cholecalciferol per day during wintertime but for 6 weeks of the intervention (total dosage 3.150 µg; 126.000 IU). They also found capsules and spray equally efficacious; mean increase in serum 25-OH-VitD levels was for about 40 nmol/L, meaning that about 80 µg (3.200 IU) of cholecalciferol was needed to increase serum 25-OH-VitD levels per 1 nmol/L.

The observed notably higher efficiency of vitamin D supplementation in our study can be explained with the fact that our intervention was conducted on participants with lower/suboptimal baseline serum 25-OH-VitD concentrations (20–50 nmol/L) while other studies also recruited subjects with sufficient vitamin D status. Therefore, baseline 25-OH-VitD levels in our study (about 40 nmol/L) were notably lower than in studies reported by Todd and by Williams (about 60 and 50 nmol/L, respectively). Baseline serum 25-OH-VitD level was previously highlighted as a probable factor affecting bioavailability of ingested vitamin D [19]. Kaykhaei et al. recently showed this consistently using considerably higher dosages (1250 µg (50.000 IU) per week; 8 weeks) of cholecalciferol [33], while Williams also noted higher efficiency of Vitamin D3 absorption in subjects with lower baseline serum 25-OH-VitD levels [32]. Although in our study the interval of baseline serum 25-OH-VitD levels was quite narrow (due to selective inclusion of subjects with suboptimal vitamin D status), we observed that the magnitude of increase in serum 25-OH-VitD concentration is higher in subjects with lower baseline levels even in this narrow interval (*p* = 0.0474, Table 3).

Another explanation for observed high efficiency of vitamin D supplementation in our study is the lower administered dosage of cholecalciferol compared to studies by Todd [31] and Williams [32]. A very recent systematic review and meta-analysis of the response of serum 25-OH-VitD concentration to vitamin D supplementation showed a very clear dose-response effect between the vitamin D dosage and serum 25-OH-VitD level [34], indicating better absorption efficiency at lower dosages. Interestingly, absorption efficiency in our study is more comparable with the intervention using 25 µg (1000 IU) buccal spray reported by Satia et al. [28]. After 4 weeks of treatment (total study dosage 750 µg; 30.000 IU) on heathy subjects (N = 7) in India, they observed an increase in serum 25-OH-VitD level for 20.0 nmol/L, meaning that about 38 µg (1.520 IU) of cholecalciferol was needed to increase serum 25-OH-VitD levels per 1 nmol/L. However, they also reported surprisingly low absorption efficiency for same dosage of vitamin D3 in the form of oil-based soft gelatin capsules (increase of serum 25-OH-VitD level was about half lower; 10.3 nmol/L). As possible explanation for this was provided by Todd et al. [35], noting genetic variability and that Asians are exhibiting lower absorption and membrane permeability than Europeans [36]. However, it should be noted that, similarly to our results, a very recent systematic review also suggested that efficiency of buccal sprays is comparable with other vitamin D3 formulations [20].

Our secondary objective was to investigate parameters other than formulation that are influencing the efficiency of absorption of vitamin D in healthy adults. Most recent meta analyses showed that baseline 25-OH-VitD concentration and age are significant indicators for supplementation increase in 25-OH-VitD level [34]. While we did not find significant age-related differences, we observed that baseline 25-OH-VitD concentration is affecting absorption efficiency, with higher efficiency at lower serum 25-OH-VitD levels. While such effect was observed previously [19], it should be noted that our study was conducted on subjects with very narrow baseline serum 25-OH-VitD concentration. Our observation further highlights the importance of baseline serum 25-OH-VitD concentration. We also found that body mass index was strongly associated with the efficiency of vitamin D supplementation (*p* < 0.0001, Table 3). Our result show that subjects with higher BMI had smaller increases in the serum 25-OH-VitD concentration. In normal body weight subjects, mean increase in serum 25-OH-VitD concentration was 35.5 ± 18.5 nmol/L, while in overweight/obese subjects, the increase was only for 21.0 ± 13.0 nmol/L. The importance of body mass index for the bioavailability of ingested vitamin D was also noted in some previous studies [19]. Jansen et al. reported that BMI should be considered for better estimation of the loading dose of cholecalciferol needed to treat vitamin D deficiency [37], but they noted that this does not exclude large interindividual variation in dose response. It is well established that obese people have higher risk for vitamin D deficiency [38,39]; Drincic et al. reported that most parsimonious explanations for this is simple dilution of 35-OH-vitD in the larger fat and tissue mass [40]. They also investigated response to graded cholecalciferol supplementation among obese adults [41], showing that, in obese patients, the dose of vitamin D should be adjusted considering the body size.

A strength of this study was that the intervention was compared with the control group and conducted during wintertime. Considering that UVB light-induced vitamin D biosynthesis is a major factor affecting serum 25-OH-VitD concentrations and that exposure of subjects to sunlight cannot be fully controlled during study, such an approach enabled us to minimize the effects of biosynthesis. Other approaches were also used to minimize the effects of vitamin D biosynthesis, including excluding traveling to areas with higher exposure to sunlight and the use of tanning beds or other artificial sources of UVB radiation. Another factor that could seriously affect results of the study are exogenous sources of vitamin D (dietary intake). Considering that it would not be reasonable to control the diet of subjects during study, a series of approaches was used to address this issue. Logically, we excluded use of supplements or medicines containing vitamin D (three months prior to inclusion and during the intervention); regular (daily) consumption of vitamin D-enriched foods, such as margarine or plant substitutes for milk/milk products; and special dietary habits. Subjects were instructed to maintain their usual diet. To assure that diet of participants is actually not very rich in vitamin D, subjects with serum 25-OH-vitD levels above 50 nmol/L were excluded. Actually, the use of predefined serum 25-OH-vitD levels as inclusion criteria also presents an important strength of our study. On one hand, this enabled us to conduct the study on vitamin D insufficient status, where supplementation is more relevant, while on the other hand, such an approach lowered interindividual variability. It should be noted that we did not include subjects with very low serum 25-OH-vitD levels (<30 nmol/L), where someone could question if this is a healthy population. It should be mentioned that response to supplementation with vitamin D could be also affected by some other factors, for example, parathyroid hormone levels and genetic parameters [42], which were not controlled in our study. Although the effects of such parameters are probably considerably smaller than of those that were controlled [43], this could be considered as a study limitation. In our study, we also observed that increase in serum 25-OH-vitD levels was associated with body mass index and baseline serum 25-hydroxyvitamin D levels. While the latter parameter was controlled, BMI was not considered during sample recruitment and group allocation, which can also be considered as a study limitation. Nevertheless, randomization resulted in a very comparable treatment/control groups with respect to BMI; therefore, we believe that this did not notably affect study results. On the other hand, having a wider interval of BMIs included turned out beneficial in the assessment of other parameters that affected the efficiency of vitamin D supplementation.

## 5. Conclusions

All three tested vitamin D formulations were efficient in improving suboptimal vitamin D status; however, two months of treatment with 25 µg (1000 IU) daily was not sufficient to assure recommended serum 25-OH-vitD levels in all subjects. We determined that efficiency of vitamin D supplementation is associated with baseline serum 25-hydroxyvitamin D concentration. Considering that the study was conducted on subjects with narrow baseline serum 25-OH-VitD concentration, such an observation further highlights the importance of baseline serum levels. We also identified that supplementation efficiency is associated with body mass index. Higher change in serum 25-OH-vitD level was observed in participants with normal body weight (BMI < 25) and in those with more pronounced vitamin D insufficiency (lower baseline 25-OH-VitD concentrations).

## Figures and Tables

**Table 1:** Descriptive statistics for subjects included to screening (N = 238).

	Serum 25-OH-VitD Concentration Avg ± SD (nmol/L)	Number of Subjects in Screening Phase N (%)				
		All Subjects	<30 nmol/L ^a^	30 to <50 nmol/L ^a,b^	50 to <75 nmol/L ^a^	≥75 nmol/L ^a^
All subjects	44.0 ± 17.0	238 (100%)	49 (21%)	107 (45%)	74 (31%)	8 (3%)
Gender:						
Male	44.3 ± 16.0	117 (49%)	21 (18%)	59 (50%)	33 (28%)	4 (3%)
Female	43.8 ± 18.0	121 (51%)	28 (23%)	48 (40%)	41 (34%)	4 (3%)
Age:						
18–29	44.3 ± 17.0	74 (31%)	14 (19%)	34 (46%)	24 (32%)	2 (1%)
30–44	44.0 ± 15.5	92 (39%)	16 (17%)	44 (48%)	31 (34%)	1 (1%)
≥45	43.5 ± 19.3	72 (30%)	19 (26%)	29 (40%)	19 (26%)	5 (7%)

Notes: ^a^ wintertime serum 25-OH-VitD concentration (at time of screening); ^b^ 107 subjects (with serum 25-OH-VitD concentration from 30 to up 50 nmol/L) meet study inclusion criteria.

**Table 2:** Descriptive statistics for subjects that completed the study (N = 99).

Variable		All Subjects	Treatment A	Treatment B	Treatment C	Treated Subjects (A + B + C)	Control Group (D)
N (%)		99	22 (22.2)	26 (26.3)	26 (26.3)	74 (74.7)	25 (25.2)
Age (SD)		37 (11)	38 (12)	35 (10)	36 (11)	36 (11)	41 (11)
Gender (%)	Male	54 (53.5)	11 (50.0)	13 (50.0)	14 (53.8)	38 (51.4)	14 (56.0)
	Female	47 (46.5)	11 (50.0)	13 (50.0)	12 (46.2)	36 (48.6)	11 (44.0)
BMI *(SD)		25.0 (4.2)	26 (4.8)	23.7 (3.9)	24.8 (4.6)	24.8 (4.5)	25.8 (3.3)
25-OH-VitD conc. Avg ± SD (nmol/L)	Baseline	39.5 (5.5)	39.5 (4.8)	39.5 (5.5)	39.0 (5.3)	39.3 (5.5)	39.5 (6.3)
	Intervention	60.3 (21.0)	70.8 (20.3)	69.0 (19.3)	66.0 (13.0)	68.5 (17.5)	35.8 (6.5)

Notes: * BMI: data missing for one subject in group A; treatments: A—vitamin D capsules with starch-adsorbed vitamin D; B—oil-based Valens vitamin D oral spray; C—water-based Valens vitamin D oral spray; and D—control group.

**Table 3:** Prevalence of the above-mean increase in serum 25-OH-VitD level and adjusted odds ratios (95% CI) in subjects included to treatment groups (N = 74).

Variable		n	Prevalence	Odds Ratio
Baseline 25-OH-VitD concentration	Bellow median	37	54.0 (37.9–69.4)	1
	At least median	37	40.5 (25.9–57.1)	0.30 (0.09–0.98) *
Intervention type	A—capsules	22	40.9 (22.6–62.2)	1
	B—oil-based spray	26	53.8 (34.8–71.9)	0.81 (0.19–3.40)
	C—water-based spray	26	46.2 (28.1–65.2)	1.11 (0.27–4.56)
Gender	Male	38	42.1 (27.4–58.3)	1
	Female	36	52.8 (36.5–68.5)	0.98 (0.32–3.05)
Age **	18–35	38	55.2(39.2–70.3)	1
	36–65	36	38.9(24.4–55.7)	0.49 (0.16–1.54)
BMI	< 25	43	69.0 (53.4–81.3)	1
	≥ 25	31	19.4 (8.8–37.3)	0.08 (0.32–3.05)*

Note: * Significant difference observed for baseline 25-OH-VitD concentration (*p* = 0.0474) and body mass index (BMI; *p* < 0.0001); ** age grouping conducted considering median age (35 years).
